# All-trans retinoic acid restores gap junctional intercellular communication between oral cancer cells with upregulation of Cx32 and Cx43 expressions in vitro

**DOI:** 10.4317/medoral.18693

**Published:** 2013-03-25

**Authors:** Juan Wang, Yaohui Dai, Yulei Huang, Xiaohua Chen, Hong Wang, Yun Hong, Juan Xia, Bin Cheng

**Affiliations:** 1 Ph.D, Department of Oral Medicine, The Guanghua School of Stomatology, Sun Yat-sen University, Guangzhou 510060, China; 2 M.M.S, Department of Oral Pathology, The Guanghua School of Stomatology, Sun Yat-sen University, Guangzhou 510060, China

## Abstract

Objective: All-trans retinoic acid (ATRA) has been demonstrated to inhibit tumor growth by restoration of gap junctional intercellular communication (GJIC) via upregulation of connexin (Cx) expression in some solid tumors. However, the relationship between ATRA and GJIC remains unclear in oral squamous cell carcinoma (OSCC). The aim of this study was to investigate the effect of ATRA on the GJIC function of OSCC. Study design: We measured the effects of ATRA on the viability and cell cycle distribution of SCC9 and Tca8113 OSCC cells. The GJIC function was observed using the scrape-loading dye transfer technique, and the mRNA and protein levels of Cx32 and Cx43 were detected by qRT-PCR, Western blot, and immunofluorescence assays. Results: ATRA inhibited the growth of OSCC cells in a dose- and time-dependent manner (P <0.05) and caused cell cycle arrest. ATRA-treated cells showed a 2.69-fold and 2.06-fold enhancement of GJIC in SCC9 and Tca8113 cells, respectively (P <0.05). Moreover, ATRA induced upregulation of Cx32 and Cx43 at both the mRNA and protein levels in OSCC cells. Conclusion: Our results indicated that restoration of GJIC via enhanced Cx32 and Cx43 expression might serve as a novel mechanism for the anti-tumor effect of ATRA in OSCC.

** Key words:**All-trans retinoic acid, oral squamous cell carcinoma, connexin, gap junctional intercellular communication.

## Introduction

Gap junctions are intercellular channels that permit the direct exchange of ions and small molecules between adjacent cells. Gap junction channels are constructed of two hemichannels (connexons) provided by each adjacent cell. These connexons are com-posed of integral plasma membrane proteins, termed connexins (Cxs). At present, approximately 21 connexin(Cx) isoforms have been characterized in the human genome (1). Gap junctional intercellular communication (GJIC) plays an important role in the maintenance of tissue homeostasis and control of cell growth and differentiation. The disruption of GJIC and abnormal expression of Cxs have been found in a series of human cancers and cell lines, including cervical carcinoma, colon cancer, and renal cell carcinoma (RCC) (2-4). Moreover, overexpression of Cx32 reduces the metastasis of RCC cells in vivo (4) and some anti-neoplastic agents were found to inhibit cell proliferation and enhance GJIC of SK-Hep-1 human hepatoma cells, which is associated with upregulation of Cx32 and Cx43 (5). These results raise the possibility that Cxs may be defined as tumor suppressors and that restoration of GJIC by induction of normal Cx expression may be a unique anti-tumor therapeutic strategy.

Among the anti-tumor agents that can restore GJIC, the vitamin A metabolite alltrans retinoic acid (ATRA) has been found to increase the amount and phosphorylation of Cx43 and enhanced GJIC in hepatoma HepG2 cells (6). Chen et al. (7) has provided evidence that ATRA can significantly restore the impaired capacity of GJIC in prostate cancer and enhanced the efficiency of cell killing during suicide gene therapy against prostate cancer. Therefore, it is necessary to explore the role of ATRA in improving GJIC of human oral squamous cell carcinoma (OSCC), the sixth ranked malignant tumor worldwide.

OSCC is the most common oral malignancy, and the 5-year survival rate of OSCC has remained at approximately 50% in spite of recent advances in diagnosis and treatment (8). Therefore, prevention and treatment of OSCC are the focus of current research. Accumulating data demonstrate that ATRA and its derivatives play an important role in both the chemoprevention and treatment of OSCC. ATRA has been previously shown to promote growth inhibition of OSCC cell lines and inhibit tumor growth in an OSCC xenograft solid-tumor model (9). However, the precise mechanism underlying the anti-tumor effect of ATRA is not yet fully understood. Previous studies have shown that OSCC growth inhibition by ATRA is mainly related to cell cycle arrest, cell apoptosis, and differentiation (10,11). Recently, Frank et al. (12) reported that human tongue squamous cell carcinoma cells were deficient in Cx43 expression. Our previous study showed that Cx43 expression decreased during 4-nitroquinoline-1-oxide–induced rat carcinogenesis (13). These results indicate that OSCC has aberrant GJIC. Moreover, studies have shown that the anti-tumor effects of ATRA on human hepatoma and prostate cancer cells are associated with restoration of GJIC function and Cxs expression (6,7). As such, modulation of GJIC may be a novel mechanism underlying the anti-tumor effects of ATRA. Therefore, we proposed that effective intervention therapy with ATRA for OSCC may be correlated with GJIC and the specific mechanisms of this action are worthy of further study. In this study, we examined the effect of ATRA on gap junction function in OSCC cells and investigated the mRNA and protein expression of Cx subtypes.

## Material and Methods

-Cell lines and cell culture

Two OSCC cell lines, SCC9 cell line (American Tissue Culture Collection, Manassas, VA, USA) and Tca8113 (Shanghai Jiao Tong University College of Stomatology, P.R. China) were routinely maintained in 1:1 mix of Dulbecco’s Modified Eagle Me-dium and Ham F12 medium (DMEM/F12) and Roswell Park Memorial Institute (RPMI)-1640 medium, respectively, supple-mented with 10% fetal bovine serum (FBS), 100 U/ml penicillin, and 100 U/ml streptomycin. Primary human normal oral epithelial (NOE) cells were harvested from the gingival mucosa of healthy patients who were undergoing impacted tooth extraction. Informed consent was obtained. Isolated NOE cells were collected using an enzymatic method (14). Cells were fed with keratinocyte serum-free medium. Cells were used for experiments at passage 3. All cells were incubated in an humidiﬁed incubator at 37° with 5% CO2. All culture reagents were purchased from Gibco (Grand Island, NY, USA).

-Reagents 

ATRA was obtained from Sigma-Aldrich (St Louis, MO, USA) and dissolved in ethanol. The concentration of ethanol was kept under 0.1% throughout all the experiments to avoid cytotoxicity.

-Cell viability analysis

Cell viability was determined by 3-(4,5-dimethylthiazol-2-yl)-2,5-diphenyltetrazoliumbromide (MTT) assay. Briefly, cells (5000 cells/well in 96-well plates) were cultured with ATRA at different concentrations (0–100μM) for 24 h and 48 h. Then, 20 μl of MTT solution (5 mg⁄ml in phosphate buffered solution; Sigma-Aldrich) was added to each well. The cells were incubated for 4 h at 37°, and the crystals were dissolved with 150 μl of dimethylsulfoxide ( DMSO, Sigma-Aldrich). The optical density (OD) value of the solution was determined at 490 nm in a microplate reader (Elx808; BioTek, Winooski, VT, USA). Cell viability was calculated according to the following formula: Cell viability (%) = OD450 (sample)/OD450 (control) ×100.

-Scrape-loading dye transfer assay

Scrape-loading and dye transfer (SL/DT) were used to measure GJIC in SCC9, Tca8113, and NOE cells. The SL/DT method has been described in detail by el-Fouly et al. (15). In brief, OSCC cells plated on 6-well plates until 70–80% confluent were treated with ATRA for 48h. Then, cells were washed twice with phosphate buffered solution (PBS) and scrape-loaded with a surgical scalpel. After 5 min incubation with 0.05% Lucifer Yellow dye (Invitrogen, Carlsbad, CA, USA) at room temperature, the dye solution was discarded and the cultures were rinsed with PBS and ﬁxed in 4% (v/v) formaldehyde. Gap junctional dye transfer was observed under a ﬂuorescent microscope (Axiovert 200; Carl Zeiss, Hamburg, Germany). Cells that received Lucifer Yellow from the scrape-loaded cells were considered communicating. The quantification of intercellular communication was accomplished by counting the numbers of cells communicating among the untreated and treated cells. GJIC was expressed as a percentage of the control (16).

-Quantitative real-time polymerase chain reaction (PCR)

Quantitative real-time PCR were performed to assay the mRNA expression of eight Cxs in OSCC cells exposed to ATRA or vehicle and NOE cells. Total RNA from cultured cells was isolated using TRIzol (Invitrogen) and reverse transcribed into cDNA with an ImProm-II reverse transcription system kit (BioRad, Hercules, CA, USA). The cDNA was analyzed by real-time PCR using a SYBR Green qPCR kit (Applied Biosystems, Foster City, CA, USA) and an Applied Biosystems (ABI) 7500 Fast PCR instrument. All primers for each gene are listed in  Table 1. PCR conditions were: 95° for 10 s followed by 40 cycles of 95° for 5 s and then 60° for 1 min. The 2-ΔΔCt method was used to quantify the relative mRNA expression using β-actin as the endogenous control.

**Table 1:**
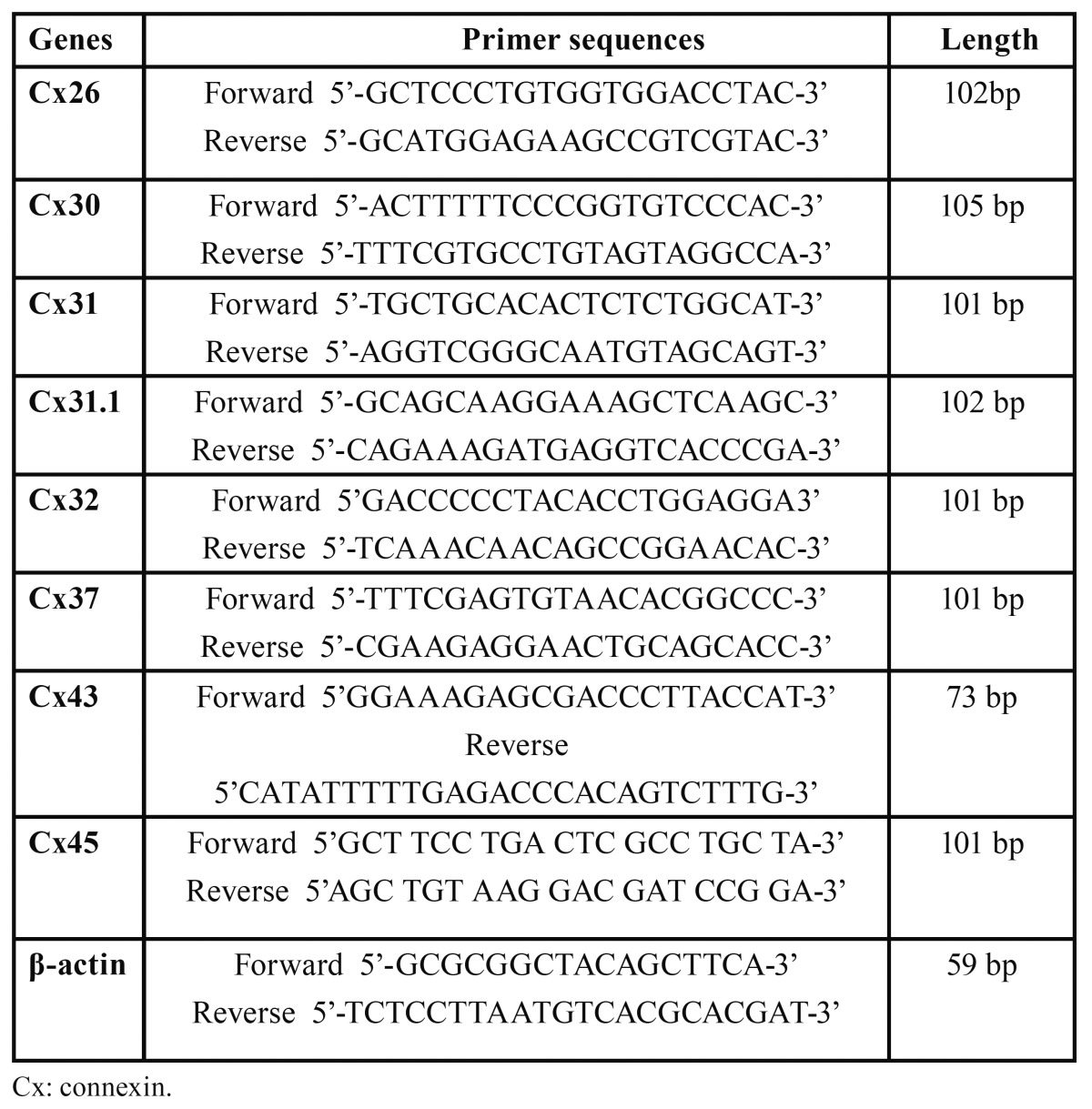
Primer sequences used in real-time PCR.

-Western blot analysis

Cells were lysed with 1% NP-40 lysis buffer (50 mM Tris, 150 mM NaCl, l mM EDTA, and 0.02% NaN3) supplemented with protease inhibitors (Roche, Indianapolis, IN, USA) for total protein extraction. The protein concentrations were measured using a BCA protein assay kit (Thermo Scientific, Rockford, IL, USA). Equal amounts of protein were loaded for SDS-PAGE and immunoblotted with the following primary antibodies: mouse monoclonal anti-Cx32 (Invitrogen; 1:800), mouse monoclonal anti-Cx43 (Invitrogen; 1:800), mouse monoclonal anti-Cx45 (Millipore, Billerica, Massachusetts, USA; 1:500), and glyceralde-hyde-3-phosphate dehydrogenase (GAPDH, Invitrogen). After incubation with horseradish peroxidase-conjugated rabbit an-ti-mouse IgG antibodies (Santa Cruz Biotechnology, Santa Cruz, CA, USA), the immunoreactive bands were detected using an enhanced chemiluminescence(ECL) detection system (Amersham Pharmacia, Piscataway, NJ, USA).

-Immunofluorescence analysis 

Immunofluorescent staining was used to verify the protein expression and examine the subcellular localization of Cx32 and Cx43. Cells were plated onto glass coverslips in six-well plates and treated with ATRA or vehicle for 48 h. Samples were fixed in 4% paraformaldehyde for 10 min, permeabilized with 0.1% Triton X-100 for 10 min, and blocked with 1% bovine serum albumin (BSA) in PBS for 30 min. Cells were incubated overnight at 4°C in 1:100 mouse anti-connexin 43 (Invitrogen) or 1:50 mouse anti-Cx32 antibody (Invitrogen). The cells were then incubated for 1 h at room temperature with rabbit anti-mouse IgG Alexa 488 (Invitrogen; 1:200). Subsequently, nuclei were counterstained with 4’,6-diamidino-2-phenylindole(DAPI)for 10 min. Samples were photographed on a fluorescent microscope (Aiovert 200; Carl Zeiss).

-Statistical analysis

Data were expressed as the mean ± standard deviation of at least three independent experiments. Statistical analysis of the results was performed using a two-tailed Student’s t-test or one-way ANOVA and post hoc multiple comparison test with SPSS software v.16.0 (SPSS, Chicago, Illinois, USA). P <0.05 was considered significant.

## Results

-ATRA inhibits cell viability 

As showed in  Table 2, ATRA (0–100 μM) induced cell growth inhibition of both SCC9 and Tca8113 cells in a dose-and time-dependent manner (P<0.05). After ATRA (20 μM) treatment for 48 h, the inhibition rate reached 53.07% in SCC9 cells and 55.01% in Tca8113 cells; at the same time, the IC50 values were 19.43±1.67 μM and 12.52±1.23 μM, respectively. The concentrations of 15 μM ATRA for SCC9 and 10 μM for Tca8113 cells were determined as optional for subsequent experiments.

**Table 2:**
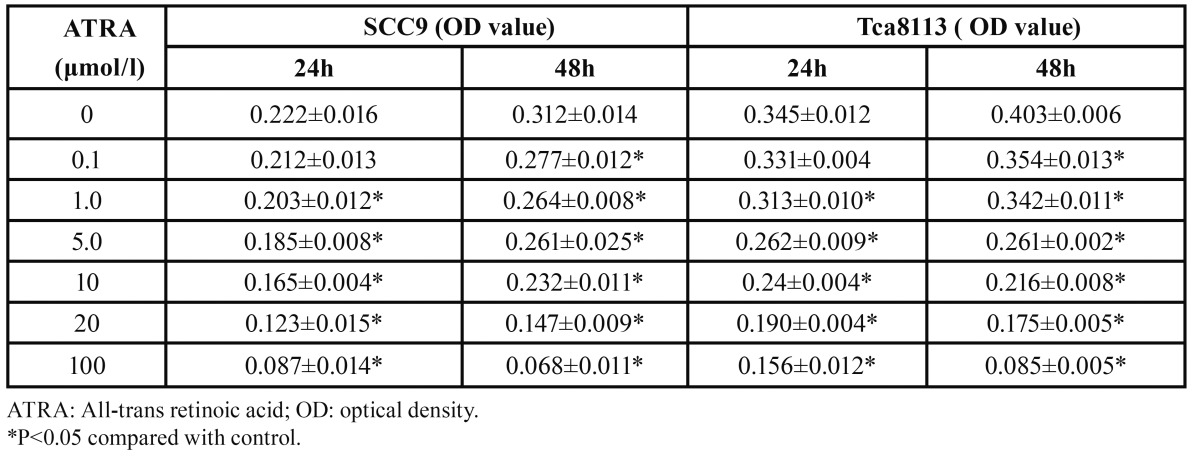
Effect of ATRA on the viability of oral squamous cell carcinoma cells.

-ATRA enhances GJIC

**Figure 1:**
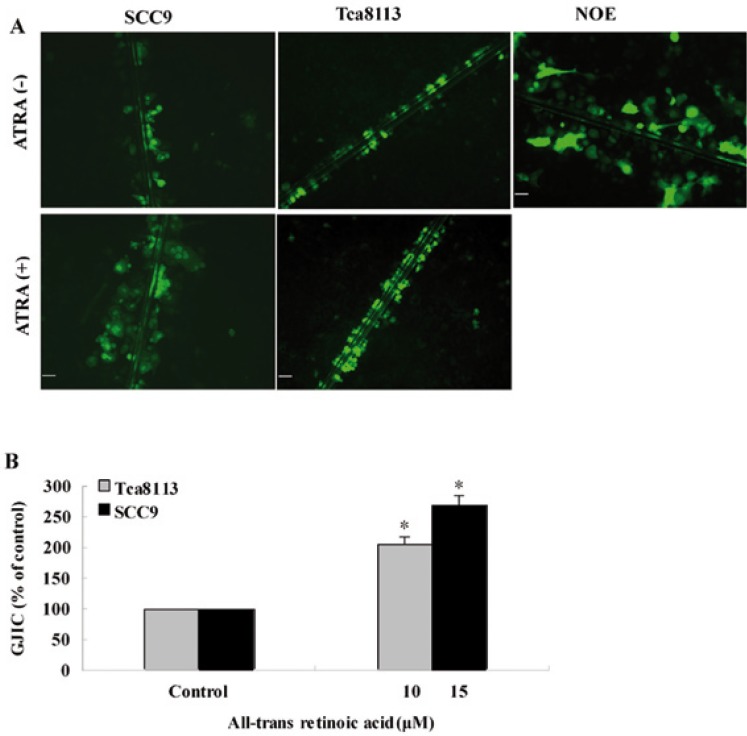
ATRA increased GJIC between oral squamous cell carcinoma cells. A) SCC9 and Tca8113 cells were treated with ATRA or vehicle for 48h, after which GJIC was assessed by the scrape-loading and dye transfer method as described in Materials and Methods. Human normal oral epithelial (NOE) cells showed at least six rows of fluorescent cells from scrape. The Lucifer Yellow fluorescent cells of SCC9 and Tca8113 were only observed in areas near to scrape. After ATRA treatment, SCC9 cells showed a significantly increased Lucifer Yellow transfer through gap junction than controls. ATRA treatment also improved GJIC of Tca8113 cells. Bar: 50μМ. B) The quantification of cells that show positive dye staining. *P < 0.05 relative to ethanol control.

In NOE cells, Lucifer Yellow was transmitted to more than six cell lines via gap junctions (Fig. 1). However, Lucifer Yellow was only visualized in cells adjacent to the scrape lines in SCC9 and Tca8113 cells. After ATRA treatment, SCC9 cells showed evident Lucifer Yellow-coupling in 4–5 layers adjacent to the scrape line. ATRA induced a 2.69-fold increase in the number of cells that showed Lucifer Yellow in SCC9 cells as compared with control-SCC9 cells(P=0.019) (Fig. 1). After exposure to ATRA, Tca8113 cells showed evident Lucifer Yellow-coupling in 3–4 layers adjacent to the scrape line. ATRA caused a 2.06-fold increase in the number of cells that were Lucifer Yellow-positive in Tca8113 as compared with control-Tca8113 cells (P=0.034).

-ATRA upregulates Cx mRNA expression 

**Figure 2:**
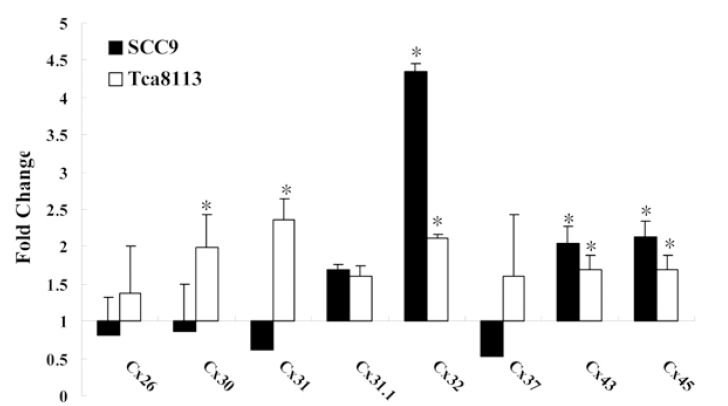
Real-time PCR analysis of the Cx mRNA expression in OSCC cells after ATRA treatment. The gene expression levels in SCC9 and Tca8113 cells were analyzed separately and were calculated in terms of fold change by using the 2-ΔΔCt equation. Colums represent the mean of three determinations for each treatment condition. Error bars represent the range factor difference (2-ΔΔCt-ΔCt SD and 2ΔΔCt+ΔCt SD). Statistical significance was calculated by Student t test; *P< 0.05 relative to control.

Real-time PCR was performed to detect the expression of eight homologous connexin isoforms: Cx26, Cx30, Cx31, Cx31.1, Cx32, Cx37, Cx43, and Cx45 (Fig. 2). ATRA significantly increased the mRNA expression of Cx32, Cx43, and Cx45 in SCC9 cells (P <0.05). Compared with control-Tca8113 cells, ATRA enhanced the mRNA expression of five Cxs in Tca8113 cells, including Cx30, Cx31, Cx32, Cx43, and Cx45 (P <0.05). However, Cx32, Cx43, and Cx45 were the three most upregulated genes induced by ATRA in both cell lines. Compared with NOE cells, both SCC9 and Tca8113 cells showed low levels of Cx32, Cx43, and Cx45 mRNA without ATRA treatment. Whereas after exposure to ATRA, the expression of the Cx32, Cx43, and Cx45 mRNAs were increased by an average of 4.34-fold, 2.03-fold, and 2.13-fold, respectively, in SCC9 cells. ATRA treatment also produced a significant increase in mRNA expression of Cx32, Cx43, and Cx45 in Tca8113 cells (2.1-fold, 1.68-fold, and 1.68-fold, respectively, as compared to the control).

Effects of ATRA on Cx protein levels and localization

**Figure 3:**
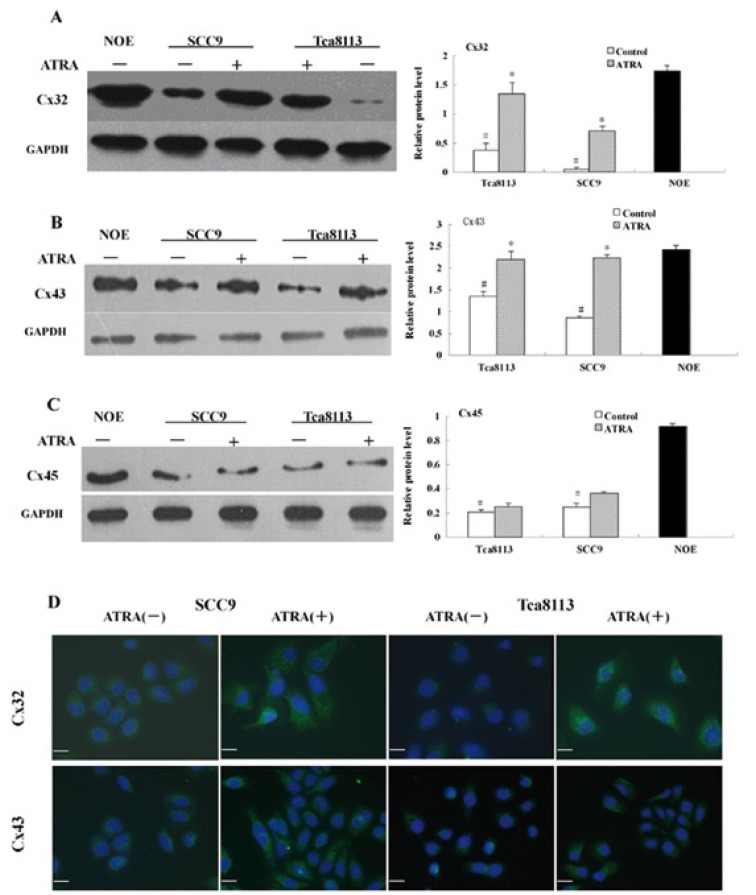
Western blot and immunofluorescence analysis of Cx32, Cx43 and Cx45 protein expression in OSCC cells after ATRA treatment. The expressions of Cx32 (A) and Cx43 (B) significantly decreased in SCC9 and Tca8113 cells as compared with human normal oral epithelial (NOE) cells. After ATRA treatment for 48h, the protein expressions of Cx32 (A) and Cx43 (B) increased in both SCC9 and Tca8113 cells. (C) Compared with human normal oral epithelial (NOE) cells, the protein expression of Cx45 also decreased in SCC9 and Tca8113 cells. However, ATRA treatment did not changed the protein level of Cx45 in SCC9 and Tca8113 cells. (D)ATRA treatment significantly increased the abundance of Cx32 and Cx43 protein and improved localization of fluorescent spots on the plasma membrane of treated SCC9 and Tca8113 cells, compared with those of controls, where the fluorescence appears scattered in the cytoplasm. #P<0.01 relative to normal oral epithelial cells; *P<0.05 relative to ethanol control. Bar: 20μМ.

Protein expression of Cx32, Cx43, and Cx45 in SCC9 and Tca8113 cells was examined by Western blot analysis. The protein levels of Cx32, Cx43, and Cx45 were decreased in both cell lines as compared with NOE cells (Fig. 3). After ATRA treatment, SCC9 cells showed a 13-fold increase in Cx32 protein levels compared with control-SCC9, while Tca8113 cells showed a 3.6-fold increase in the Cx32 protein (Fig. 3). ATRA also induced a 2.6-fold and 1.6-fold increase of Cx43 protein expression in SCC9 and Tca8113 cells, respectively (Fig. 3). However, both SCC9 and Tca8113 cells displayed no significant change in Cx45 protein concentration after ATRA treatment (Fig. 3).

The subcellular localization of Cxs was examined by immunofluorescence assay. In treated SCC9 cells, green fluorescence was located throughout the cell membrane and cytoplasm and the Cx32 and Cx43 staining increased and localized at the cell mem-brane. In contrast, untreated cells displayed a small number of positive spots and diffuse cytoplasmic staining for Cx32 and Cx43. In Tca8113 cells, ATRA treatment produced similar findings to those observed in SCC9 cells and increased the mem-brane-association and levels of Cx32 and Cx43 as compared with control-Tca8113 cells (Fig. 3).

## Discussion

ATRA, an active and natural derivative of vitamin A, plays an important role in many physiological functions, such as growth, differentiation, reproduction, apoptosis, homeostasis, and fetal development (17). In clinical studies, retinoids have been shown to have a promising role in the chemoprevention and chemotherapy of human head and neck cancers. ATRA has been used to control oral lesions of lichen planus as well as to prevent and reverse the progression of oral leukoplakia to malignant tumors. It has also shown an anti-tumor therapeutic effect on OSCC (18,19). The mechanisms of ATRA action are not completely understood, although several mechanisms have been reported, including modulation of differentiation and proliferation, induction of apoptosis and cell cycle arrest, and downregulation of telomerase activity (20,21). Recently, several studies have demonstrated that ATRA has the ability to modulate GJIC in some cancers, and alteration of GJIC may be a new mechanism for the chemopreventative/chemotherapeutic actions of ATRA. However, there is little evidence for the effect of ATRA on the GJIC in human OSCC cells. In the present study, we demonstrated that ATRA significantly inhibited the cell viability in two OSCC cell lines, SCC9 and Tca8113. Moreover, ATRA enhanced the functional gap junctions of OSCC cells. To the best of our knowledge, it is the first report regarding the effect of ARTA on GJIC in human OSCC.

Recent studies have demonstrated that aberrant GJIC is an important step in the carcinogenesis process. Moreover, GJIC restoration in tumor cells contributes to reversing the transformed phenotype and increases cell growth control both in vitro and in vivo. Therefore, prevention of GJIC downregulation and restoration of GJIC in tumor cells could be a rational chemopreventive approach (22). ATRA, trans-resveratrol, growth factors, and green tea components have a positive affect on GJIC. These compounds might confer their cancer prevention attributes by upregulating Cxs and GJIC (23). Watanabe et al. (24) demonstrated that ATRA deregulates GJIC and prevents the disruption of GJIC in vitro in renal epithelial cells exposed to renal carcinogens. Furthermore, ATRA increases the expression of the Cx43 protein at the plasma membrane. These results indicate that one mechanism underlying the anti-tumor effect of ATRA in renal epithelial cells is the recovery of GJIC function via the enhanced expression and normalized distribution of the Cx43 protein. In human hepatoma HepG2 cells, ATRA exerts antiproliferative, differentiative, and apoptotic effects. Furthermore, ATRA increases the amount and phosphorylation of Cxs and enhances GJIC. This suggests that ATRA might be able to reverse cell transformation in HepG2 cells (6). In this study, we found that ATRA could enhance functional gap junctions in OSCC cells. Our data is consistent with above reports that show ATRA upregulates GJIC in cell systems in vitro. However, previous studies have also shown that ATRA impairs GJIC in some cell types. ATRA decreases the expression of the Cx43 protein and functional gap junctions during the neuronal differentiation of p19 embryonic carcinoma cells and human pluriopotential teratocarcinoma cells (25,26). George et al. (27) also found that ATRA inhibits both cell growth and GJIC at similar doses in human cutaneous squamous cell carcinoma SCC-13 cells. However, to date, the mechanisms responsible for these cell-type discrepancies are unknown, though they may be relative to cell-type–specific transcriptional regulators. In addition, specific Cx expression patterns are variable. It is possible that a particular Cx may function as a tumor modulating protein in one or several specific cell types but not in others. More systematic studies are needed to explore the precise mechanisms underlying ATRA’s effects on GJIC in cancer.

According to previous studies and our present findings, we propose that ATRA can restore GJIC to some extent. However, further studies are required to understand the mechanisms by which ATRA alters GJIC in OSCC cells. The connexin family comprises the principal membrane components of gap junctions. Connexin proteins are expressed in a tissue- and cell-type specific manner. In oral mucosal epithelium, although Cx43 and Cx26 are present (28), other subtypes of Cx have not yet been examined. Thus, we used qRT-PCR analysis to evaluate the expression of eight Cxs in two OSCC cell lines. Our results showed that normal oral epithelial cells had good GJIC capability; however, the communication between OSCC cells was defective and the levels of the Cx32 and Cx43 proteins were decreased. To our knowledge, there is little evidence concerning Cx32 expression in oral carcinogenesis. In this study, we first demonstrated that Cx32 was downregulated in human OSCC cells. Our results are consistent with previous reports that indicate aberrant gap junction function is correlated with carcinogenesis, suggesting that deficiency of GJIC function may be associated with oral carcinogenesis similar to other types of cancers. Next, we measured the effect of ATRA on the expression of Cx32 and Cx43. In our study, qRT-PCR and Western blot analysis demonstrated that ATRA induced a marked increase in Cx32 mRNA and protein expression. Fluorescent analysis of Cx32 showed that the protein was partially localized to the plasma membranes between contacting cells. This result suggested that ATRA may also influence Cx32 relocalization and positioning in the cell membrane of OSCC cells. At the current time, there is little evidence for an effect of ATRA or other anti-neoplastic agents on Cx32 expression in OSCC cells. It has been reported that ATRA enhances Cx32 expression in some cell types. For instance, ATRA treatment increases Cx32 expression and improves the localization of Cx32 to the cell membrane in human hepatocellular carcinoma deficient in the expression of Cx32 (6). Other antineoplastic agents have also been shown to restore GJIC by upregulating Cx32 expression in some cancer cells. Fucoxanthin rich in carotenoids inhibits cell proliferation and enhances GJIC in SK-Hep-1 human hepatoma cells, and these effects are associated with upregulation of the Cx32 and Cx43 proteins (5). Collectively, these results indicate that one possible mechanism for the anti-cancer effect of ATRA in OSCC is the recovery of GJIC function by enhanced transcriptional expression and normalized distribution of the Cx32 protein.

In addition, our studies showed that ATRA treatment induced a significant enhancement in protein and mRNA expression of Cx43 in OSCC cells, consistent with previous reports in some human cancer cells and lens epithelial cells (29) in which the levels of GJIC and Cx43 expression were increased by ATRA treatment. Indeed, several other reports have also demonstrated that Cx43 can be induced in OSCC cells. Livny et al. found that that lycopene, an anticarcinogenic agent, significantly upregulated both the transcription and the expression of Cx43 and enhanced gap junctional communication between oral cancer cells (30). Taken together, these results suggested that transcriptional regulation is involved in Cx43 expression in oral cancer cells and that ATRA may restore functional GJIC through transcriptional regulation of Cx43 expression in OSCC cells.

Our data showed that ATRA restored GJIC and promoted the mRNA and protein expression of Cx32 and Cx43, which is de-creased in OSCC cells. These findings indicated that a novel mechanism for the anti-tumor effect of ATRA in OSCC cells is restoration of GJIC function by enhancing Cx32 and Cx43 expression, although the exact mechanisms still require further studies. Restoration of gap junctional communication by ATRA may provide new perspectives for ATRA application in human oral cancer therapy.
